# Impact of Endometallofullerene on P84 Copolyimide Transport and Thermomechanical Properties

**DOI:** 10.3390/polym10101108

**Published:** 2018-10-07

**Authors:** Galina Polotskaya, Maia Putintseva, Alexandra Pulyalina, Iosif Gofman, Alexander Toikka

**Affiliations:** 1 Institute of Chemistry, Saint Petersburg State University, Universitetskiy pr. 26, Saint Petersburg 198504, Russia; g_polotskaya@mail.ru (G.P.); a.toikka@spbu.ru (A.T.); 2 Institute of Macromolecular Compounds, Russian Academy of Sciences, Bolshoy pr. 31, Saint Petersburg 199004, Russia; gofman@imc.macro.ru; 3 A.V. Topchiev Institute of Petrochemical Synthesis, Russian Academy of Sciences, Leninsky Prospect 29, Moscow 119991, Russia; hippopotable@mail.ru

**Keywords:** membrane, pervaporation, nanomodifiers, methanol—methyl acetate mixture, endometallofullerene, copolyimide

## Abstract

Novel polymer composite materials, including unique nanoparticles, contribute to the progress of modern technologies. In this work, the endohedral fullerene C_60_ with incapsulated iron atom (endometallofullerene Fe@C_60_) is used for modification of P84 copolyimide. The impact of 0.1, 0.5, and 1 wt % endometallofullerene on the structure and physicochemical properties of polymer films is studied through scanning electron microscopy, thermogravimetric analysis, and thermomechanical tests. Transport properties are estimated through sorption and pervaporation techniques toward methanol and methyl acetate mixture. The inclusion of endometallofullerene into the copolyimide matrix improves membrane permeability and selectivity in the separation of methanol—methyl acetate mixtures. The maximal effect is achieved with a composite containing 0.5 wt % Fe@C_60_. The developed composites are effective for energy and resource saving purification of methyl acetate by pervaporation.

## 1. Introduction

The development of novel composite materials through polymer modification with nanoparticles promotes the progress of modern technologies, such as membrane gas and liquid separation [1,2,3]. Innovative membrane materials with unique physicochemical and transport properties have been created with the use of carbon nanoparticles (nanotubes, fullerenes, graphene and its derivatives) as a modifier [4]. One of the attractive properties of hollow carbon clusters, known as fullerenes, is the possibility to use them as robust containers for other species [5]. The term “endohedral” is nowadays used for fullerenes with species (atoms, ions, or molecules) incorporated into the carbon cage [6]. Endohedral fullerene with incorporated atom of metal (endohedral metallofullerene or endometallofullerene) has attracted great attention since 1990 [7]. The structural, chemical, and electronic properties of endometallofullerenes are different from those of empty fullerenes. The chemical activity of fullerenes is mostly determined by their π-system. In endometallofullerene, the π-system of fullerene is modified by the electron transfer from the metal atom and by an inhomogeneous distribution of excess electron density over the fullerene surface. This raises an intriguing question of the mutual influence of the endohedral atoms on the addition pattern and physicochemical properties of the endometallofullerene derivatives [8,9]. 

Applications of endohedral fullerenes with encapsulated rare elements (Gd, Ho, Dy, Nd, and so on) in organic photovoltaics [10], and especially in biomedicine, have been extensively reviewed [11,12,13]. 

Endohedral fullerene C_60_ with incapsulated iron atom (Fe@C_60_) has been recently obtained [14]. The Fe@C_60_ nanoparticles have been used as a filler of the poly(2,6-dimethyl-1,4-phenylene oxide) (PPO) matrix for membrane applications. These membranes were effective in O_2_/N_2_ and He/N_2_ separation and exhibited high selectivity in the pervaporation of water—ethyl acetate mixtures [15].

**Figure 1:**
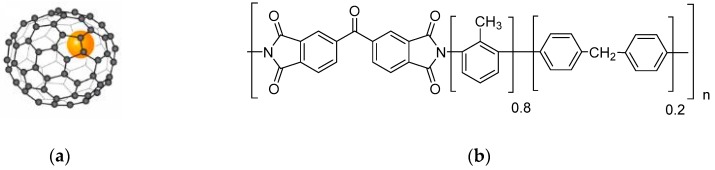
Structure of (**a**) endometallofullerene Fe@C_60_ and (**b**) P84 copolyimide.

In the present work, Fe@C_60_ nanoparticles are used for the modification of P84 copolyimide synthesized from dianhydride 3,3′-4,4′-benzophenone tetracarboxylic acid and two diamines: Metaphenylene diamine (80%) and diaminodiphenylmethane (20%). Figure 1 shows the endometallofullerene Fe@C_60_ and P84 copolyimide structure.

P84 is a commercially available copolyimide, which exhibits good mechanical properties, chemical resistance, and low hydrophilic properties; it has been studied as a membrane material for ultrafiltration [16], nanofiltration [17], and gas separation [18]. Chung et al. agreed that P84 is a prospective membrane material for the pervaporation dehydration of water‒alcohol mixtures containing ethanol, isopropanol, and tert-butanol [19,20,21]. The modification of a P84 membrane with different particles increases efficiency of isopropanol dehydration: Including zeolite 5A and 13X (up to 20 wt %) increases permeability and selectivity compared to pure P84 [22]; including 20 wt % ZIF-90 particles leads to a more than double increase of flux, while selectivity doesn’t change [23].

The aim of the present work is the development of novel thin film membranes through modification of copolyimide P84 with Fe@C_60_ nanoparticles (0.1, 0.5, and 1 wt %) and the study of the membrane structure, thermomechanical properties, and transport parameters in separation of methanol and methyl acetate mixture by pervaporation. 

Separation of methanol—methyl acetate mixtures is an important industrial task, since these substances are widely used in the chemical industry as reactants or solvents [24,25,26,27,28]. For example, both methanol and methyl acetate are involved in the synthesis of acetic acid and acetic anhydride; production of poly(vinyl alcohol) occurs through the alcoholysis of poly(vinyl acetate) in a methanol solution; a byproduct of this process is methyl acetate [29].

In the industry, butyl acetate is produced through a transesterification reaction of methyl acetate with *n*-butanol, which leads to the formation of butyl acetate and methanol.

Methyl acetate (MeOAc) + n-butanol (BuOH) ↔ n-butyl acetate (BuOAc) + methanol (MeOH)

To provide the efficient occurrence of this equilibrium reaction and to optimize the process by shifting chemical equilibrium, it is necessary to remove methanol. Unfortunately, this task is complicated by the fact that unreacted methyl acetate forms azeotropic mixtures with methanol: 18 wt % methanol and 82 wt % methyl acetate at 760 mm Hg [30]. Separation of azeotropic mixtures by pervaporation technology is a convenient, eco-friendly, and cost-effective method.

## 2. Materials and Methods

### 2.1. Materials

P84 copolyimide (BTDA–TDI/MDI) was purchased from HP Polymer GmbH (Lenzing, Austria). The polymer was dried overnight at 120 °C under vacuum before use. The nanoadditive Fe@C_60_ as 1.5 wt % solution in *N*,*N*-dimethylformamide was provided by PINP, Kurchatov Institute Russian Research Center (Moscow, Russia). Reagent-grade methanol, methyl acetate, and *N*,*N*-dimethylformamide (DMF) manufactured by Vekton (Saint Petersburg, Russia) were used without further purification.

### 2.2. Membrane Preparation

P84/Fe@C_60_ composites comprising 0.1, 0.5, and 1 wt % Fe@C_60_ were prepared by mixing calculated amounts of a 10 wt % P84 solution and a 1.5 wt % Fe@C_60_ solution in DMF at room temperature under stirring for 2 h and then held in an ultrasonic bath at 40 °C for 40 min until reaching complete homogenization; after that, the solution was filtered to remove any mechanical impurities.

The membranes were prepared by casting 10 wt % solutions of P84 or P84/Fe@C_60_ in DMF onto a glass plate. The solvent was removed by evaporation at 40 °C; the membranes were separated from the support and dried in a vacuum oven at 60 °C to constant weight. The resulting films had a thickness of ~20 μm.

### 2.3. Sorption Test

Sorption studies were conducted by immersing the samples into individual liquids (methanol, methyl acetate) at atmospheric pressure and a temperature ~20 °C. At certain intervals, the samples were removed and weighed on an analytical balance with an accuracy of ±10^−4^ g. The experiment was continued until the achievement of a state of equilibrium (two months). Sorption degree, *S* (%) was calculated using the formula:

(1) $$S=\left[\left(m-m_{0}\right)/m_{0}\right]\cdot 100,$$

where *m*_0_ is the initial weight of the sample and *m* is the weight of the swollen sample.

### 2.4. Pervaporation Test

The pervaporation performances of the membranes under study were examined with the stainless steel laboratory cell with stirring at 50 °C and atmospheric pressure. Downstream pressure below 10^−2^ mm Hg was maintained. Detailed description of the experimental procedure is described in [31,32,33]. The membrane effective area was 14.8 cm^2^. The permeate was a methanol—methyl acetate mixture, with a methanol content of 5 to 25 wt %. The obtained data were used to calculate the process parameters of the membranes [34].

The total flux through the membrane (*J*) was determined as the amount of liquid penetrated through membrane area per time unit. In order to compare membranes of different thickness (varying from 15 µm to 23 µm), the flux value was normalized to the corresponding flux through a membrane of 20 µm thickness. The normalized flux *J_n_* was calculated as:

(2) Jn=J·l20,

The separation factor (*α_MeOH/MeOAc_*) was defined as follows:

(3) $$\alpha_{MeOH/MeOAc}=\left(Y_{MeOH}/Y_{MeOAc}\right)/\left(X_{MeOH}/X_{MeOAc}\right),$$

where *X_MeOH_*, *X_MeOAc_*, and *Y_MeOH_*, *Y_MeOAc_* are the weight fractions of methanol and methyl acetate in the feed and in the permeate, respectively.

The pervaporation separation index (PSI) [32], which is the index of the separation effectivity of a membrane, has been defined based on the total flux and separation factor:

(4) PSI=J·(α−1),

In order to establish the effect of the membrane nature, excluding the operating conditions on separation properties, the permeability and selectivity were calculated [35,36]. Membrane permeability (*P_i_*, Barrer) was obtained using the equation:

(5) $$P_{i}=j_{i}l/\left(p_{io}-p_{il}\right),$$

where *j_i_* is a molar flux of component *i*, cm^3^ (STP)/cm^2^ s, and *p_i_*_0_ and *p_il_* are the partial pressures of component *i* on both sides of the membrane (0 stands for the surface on the feed side and *l*—for the surface on the feed side).

Membrane selectivity *β_MeOH/MeOAc_* was defined as the ratio of the permeabilities:

(6) βMeOH/MeOAc=PMeOHPMeOAc,

### 2.5. Membrane Characterization

A scanning electron microscope SEM Zeiss SUPRA 55VP (Carl Zeiss AG, Oberkohen, Germany) was used to study the morphology of the membrane cross-sections. The membrane cross-sections were produced by cleaving the membranes in liquid nitrogen. After that, the surface samples were covered by a gold layer via cathode sputtering using the Quorum 150 (Quorum Emitech, Ashford, UK) installation.

Mechanical characteristics of the films, such as the Young’s modulus *E*, yield stress *σ_y_*, tensile strength *σ_b_*, and ultimate strain *ε_b_* values were obtained using bandlike samples of 2 mm width and 20 mm length. The experimental technic is described in [37].

The thermogravimetric analysis of the film samples was conducted in the micro-thermobalance TG 209 F3 Iris (NETZSCH, Selb, Germany) under the conditions of sample heating from room temperature up to 600 °C in the self-generating atmosphere with a speed of 5 deg/min. The indices of thermal stability of the films were determined through thermomechanical tests.

The glass transition temperatures *T_g_* of the samples were determined through thermomechanical tests using the TMA 402 F1 Hyperion (NETZSCH, Selb, Germany) test system. The heating speed of the samples under the action of a constant extension load of 40 kPa was 5 deg/min.

## 3. Results

The compatibility between the polymer matrices and inorganic fillers plays an important role, and extensive research has been conducted to support the proper selection of parent pairs for composite materials [38,39,40]. Both components of the present composite, P84 polymer and Fe@C_60_ inorganic filler, are solved in the same solvent DMF, which facilitates the procedure of these components mixing in solution to prepare nondefective membranes.

**Figure 2:**
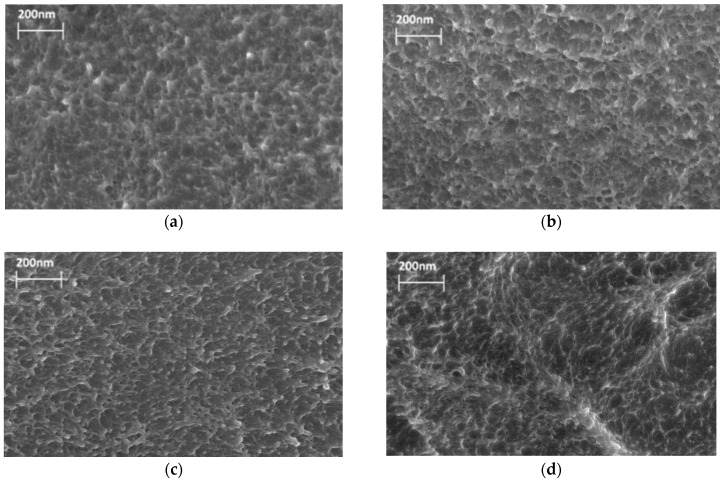
SEM micrographs of membrane cross-section: (**a**) P84, (**b**) P84/Fe@C_60_(0.1%), (**c**) P84/Fe@C_60_(0.5%), and (**d**) P84/Fe@C_60_(1%).

Membrane morphology was studied through SEM. Figure 2 shows cross-section micrographs of the P84 membrane modified with different amounts of the filler: 0.1, 0.5, and 1 wt % Fe@C_60_. The cross-section of the P84 membrane (Figure 2a) has a homogeneous structure with small (≤0.1 μm) elements of supramolecular structures. The morphology of the membranes containing 0.1 and 0.5 wt % Fe@C_60_ (Figure 2b,c) does not essentially differ from that of pure P84. This fact indicates the advantages of composite preparation using the solution technique and the good compatibility of Fe@C_60_ and P84 matrix at low concentration of filler. The P84/Fe@C_60_(1%) membrane exhibits a slightly damaged structure that may be connected to an excess of the modifier randomly distributed in the matrix.

### 3.1. Mechanical and Thermal Properties

The results of the mechanical tests and values of the glass transition temperature for the films of P84 and its composites containing up to 1 wt % Fe@C_60_ are presented in Table 1. The inclusion of Fe@C_60_ nanoparticles into the P84 matrix does not affect the qualitative character of the stress–strain behavior of the materials: In all cases a well-defined stress maximum, a yield point can be seen at the stress-strain curves; during further deformation, all samples tend to form a neck. The break of the films takes place just in the necking zone. Both *E* and *σ_y_* values are unaffected by the variation of Fe@C_60_ content in the material. 

**Figure 3:**
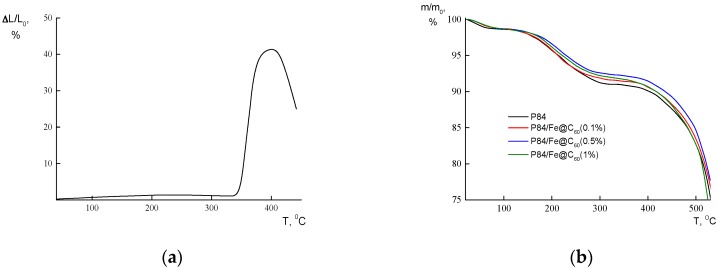
(**a**) Thermomechanical curve: The deformation of the sample (Δ*L/L*) vs. temperature; (**b**) TGA curves of P84 and P84/Fe@C_60_ membranes.

The thermomechanical tests showed the realization of only one thermally stimulated transition, with the glass transition in the temperature range of 344–346 °C for all films (Figure 3a). Only a very weak increase of *T_g_* value was registered as a result of the inclusion of Fe@C_60_ into the P84 matrix (Table 1). A substantial decrease in the rigidity of all films studied was registered in the temperature range above *T_g_* (the increase of the deformation value of the film sample along with the sample heating under a constant extension load). The speed of the thermally stimulated elongation of the samples in the temperature range 350–370 °C was as high as 1.5%/degree. However, the substantial fall of the deformation speed was registered while heating the membrane samples from 370 up to ~400 °C; at 400 °C, the sample’s elongation ceases and above this temperature, up to 430 °C, the rigidity of the material tends to increase along with the increase of the temperature: The thermally stimulated sample’s contraction can be seen in this range of temperatures in Figure 3a. This behavior at high temperatures testifies to the onset of the thermal destruction process in the materials that provokes the formation of the interchain crosslinks (so called “destruction induced crosslinks”).

Table 2 lists the thermal stability indices of the membranes, that is, the temperatures (*τ*_1_, *τ*_5_, and *τ*_10_) at which the sample weight reduces in 1, 5, and 10 wt %, respectively, under the thermal destruction processes. These data were obtained through TGA tests (Figure 3b). It was shown that the inclusion of Fe@C_60_ into the copolyimide matrix does not provoke any substantial variations of thermal stability of the materials: Only a modest increase of the *τ*_1_, *τ*_5_, and *τ*_10_ values of the films (within ~10 degrees) was registered for the samples containing 0.1–0.5 wt % filler.

### 3.2. Transport Properties

Mass transfer through P84/Fe@C_60_ thin film membranes was studied with regard to two organic liquids: Methanol and methyl acetate, using sorption and pervaporation tests. Table 3 lists some physical properties of liquids under study to predict the behavior of substances during mass transfer. Methanol and methyl acetate have similar boiling points and density, but the molecular volume, viscosity, and solubility parameter of these substances are significantly different. The solubility parameter *δ* is used to evaluate the interaction of the polymer with the liquid. According to solubility theory [41], the smaller the difference between solubility parameters |Δ*δ*| of the polymer and the liquid substance is, the better the solubility of this substance in the polymer. The solubility parameter *δ* of pure P84 is 22.3 (J/cm^3^)^1/2^ [42]. Thus, it is assumed that methyl acetate solubility (|Δ*δ*| = 2.7) should be preferential compared to methanol (|Δ*δ*| = 7.4).

Sorption experiments were carried out by immersion of membrane samples into the individual liquid (methanol or methyl acetate) at atmospheric pressure and temperature of 25°C. It is known [19] that the process of P84 swelling is quite long, so the experiment lasted two months. The obtained values of the sorption degree are presented in Table 4. The sorption degree of methyl acetate is higher than that of methanol for all membranes under study; this result agrees with the close values of the solubility parameters of methyl acetate and P84. With the increase of the Fe@C_60_ content in the membrane, the sorption degree of methanol increases substantially, while the sorption degree of methyl acetate decreases slightly.

These results will have an effect on the separation properties of membranes in the pervaporation of methanol—methyl acetate mixtures.

### 3.3. Pervaporation of Methanol—Methyl Acetate Mixture

**Figure 4:**
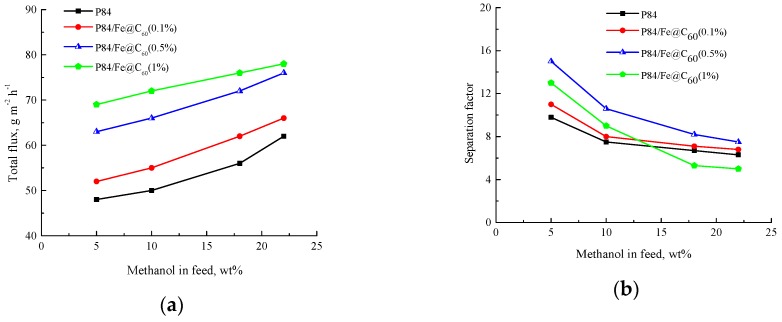
Dependence of (**a**) total flux and (**b**) separation factor *α_MeOH/MeOAc_* on methanol concentration in the feed for the pervaporation of the methanol—methyl acetate mixture through membranes based on P84 and P84/Fe@C_60_, 25 °C.

Membranes based on P84 and modified with different amount of Fe@C_60_ were studied in the pervaporation of the methanol—methyl acetate mixture at 5–22 wt % methanol concentration in the feed, including the azeotropic mixture. The permeate was considerably enriched with methanol for each composition of the feed. Figure 4a shows the dependence of total flux through membranes on the methanol concentration in the feed. The total flux through membranes increases with increasing methanol concentration. An increase of Fe@C_60_ content in the membrane results in an increase of the total flux.

Figure 4b shows the dependence of the separation factor *α_MeOH/MeOAc_* on the methanol concentration in the feed. For all membranes, the separation factor decreases with increasing methanol concentration. The membrane containing 0.5 wt % Fe@C_60_ exhibits the best separation capacity. The separation factor for the P84/Fe@C_60_(1%) membrane is unstable, and its value is lower than that of the original P84 membrane. 

This result is undoubtedly connected with the negative effect of excessive endometallofullerene content in the composite and correlates with the data obtained through SEM (Figure 2) that show the slightly damaged structure of the P84/Fe@C_60_(1%) membrane, with lower mechanical and thermal properties (Table 2 and Table 3), compared to those of the P84 membrane.

**Figure 5:**
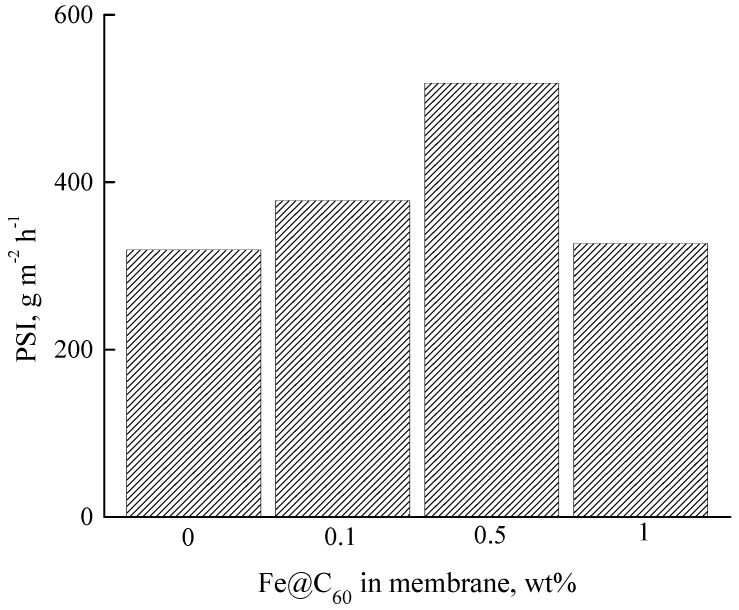
Dependence of the pervaporation separation index *(PSI)* on Fe@C_60_ content in the membrane in the pervaporation of the methanol—methyl acetate (18:82 wt %) azeotropic mixture, 25 °C.

Particular attention was given to separation of the methanol—methyl acetate (18:82 wt %) azeotropic mixture by pervaporation, using membranes based on P84 and P84/Fe@C_60_. The efficiency of the membranes in the separation of the azeotropic mixture was estimated using the pervaporation separation index (*PSI*), which includes both the total flux and separation factor of the membrane (Equation (4)). Figure 5 shows the dependence of the *PSI* on the Fe@C_60_ content in the membrane; it is evident that the best membrane for this purpose is the membrane containing 0.5 wt % Fe@C_60_ in the P84 matrix.

**Figure 6:**
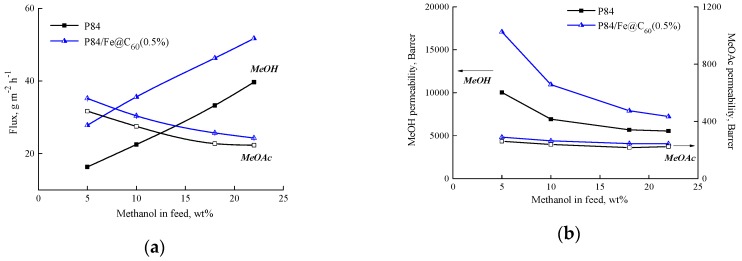
Dependence of (**a**) flux and (**b**) permeability of individual components (methanol and methyl acetate) on methanol concentration in the feed for the pervaporation of the methanol—methyl acetate mixture through P84 and P84/Fe@C_60_(0.5%) membranes, 25 °C.

**Figure 7:**
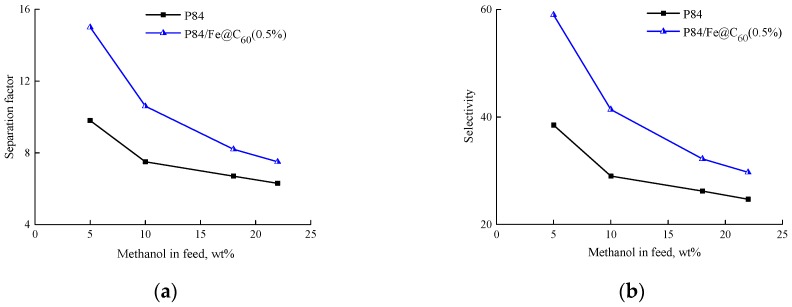
Dependence of (**a**) separation factor *α_MeOH/MeOAc_* and (**b**) selectivity *β_MeOH/MeOAc_* on methanol concentration in the feed for the pervaporation of the methanol—methyl acetate mixture through the P84 and P84/Fe@C_60_(0.5%) membranes, 25 °C.

Modern scientific literature recommends describing the transport properties of membranes in pervaporation processes, not only in terms of flux and separation factor, but also in terms of selectivity and permeability [44,45]. The calculation of permeability and selectivity makes it possible to exclude the driving forces of the separation process (partial vapor pressure) and to reveal the characteristic properties of the membrane–penetrant system. The permeabilities of individual substances (methanol and methyl acetate) and selectivity *β_MeOH/MeOAc_* for the original P84 membrane and the best modified P84/Fe@C_60_(0.5%) membranes were calculated according to the procedures described by Baker et al. [35]. Figure 6 and Figure 7 present these parameters in comparison with the data on the flux and separation factor for the P84 and P84/Fe@C_60_(0.5%) membranes.

Figure 6a shows the dependence of individual components’ (methanol and methyl acetate) flux on methanol concentration in the feed. The flux of methanol increases and the flux of methyl acetate decreases with increasing methanol concentration in the feed. When the driving force contribution is removed (Figure 6b), the permeability of methanol decreases, and the permeability of methyl acetate changes slightly with increasing methanol concentration in the feed. Membrane P84/Fe@C_60_(0.5%) shows a greater affinity for methanol and methyl acetate, compared to the P84 membrane. The curve of the methanol permeability has a course that is the opposite to that of the methanol flux, that is, methanol permeability decreases with increasing methanol concentration in the feed. It means that the increase of flux mainly occurs due to an increase in the driving force (partial pressures) of penetrating liquids.

Figure 7 shows the dependence of the separation factor *α_MeOH/MeOAc_* and selectivity *β_MeOH/MeOAc_* on methanol concentration in the feed. With increasing methanol content in the feed, selectivity decreases, as well as the separation factor. The P84/Fe@C_60_(0.5%) membrane is characterized by higher values of the separation factor and selectivity in comparison with the P84 membrane.

The transport properties of the P84/Fe@C_60_(0.5%) membrane developed in this work were compared with literature data for the case of the pervaporation separation of the methanol—methyl acetate mixture close to azeotrope. Table 5 summarizes the results obtained for different polymers and feed compositions from a number of papers [29,46,47,48]. These data show that the separation factor of the P84/Fe@C_60_(0.5%) membrane significantly exceeds this parameter for other membranes except PA/ND (3%) [47]. However, the flux of the last two membranes is much lower than that of the Pervap 2255 membranes. This difference can be explained by the bilayer structure of Pervap 2255 having a thin selective layer (3–5 μm) supported on a porous substrate, whereas our membranes have a greater thickness (~20 μm). The creation of composite membranes with a thinner selective layer P84/Fe@C_60_(0.5%) is a promising task for subsequent studies. It should be noted that there is the possibility of improving the performance of P84/Fe@C_60_(0.5%) membranes, probably through the development of bilayer composite membranes or other modes, and this will be the subject of further research.

## 4. Conclusions

In the present work, composite membranes based on copolyimide P84 with different contents (0.1, 0.5, and 1 wt %) of the new modifier—endohedral fullerene C_60_ with an incapsulated iron atom were developed. The structure of the membranes was studied through scanning electron microscopy; it was shown that the matrix polymer and the filler are well compatible at low concentrations of nanoparticles. Deformation-strength and thermomechanical tests showed that the P84/Fe@C_60_ membranes have mechanical characteristics that are sufficient for practical use. An excessive endometallofullerene content manifests in a slight deterioration of the structure and thermomechanical properties of the P84/Fe@C_60_(1%) membrane. The transport properties of the resulting membranes were studied using sorption and pervaporation tests. When endometallofullerenes are included into the P84 matrix, the sorption degree increases, and the main parameters of mass transfer, namely, the total flux and the separation factor, improve. The maximal effect from the introduction of nanoparticles is achieved for a membrane containing 0.5 wt % Fe@C_60_. With a further increase in the modifier content, the separation factor sharply decreases. Thus, the inclusion of Fe@C_60_ nanoparticles into the P84 matrix has a positive effect on the transport properties of membranes and requires further study.

## Figures and Tables

**Table 1:** Mechanical properties and glass transition temperature of membranes.

Membrane	*E*, GPa	*σ_y_*, MPa	*σ_b_*, MPa	*ε_b_*, %	*T_g_*, °C
P84	2.41 ± 0.09	113 ± 2	113 ± 2	7.4 ± 0.4	344
P84/Fe@C_60_(0.1%)	2.40 ± 0.07	112 ± 4	109 ± 4	7.8 ± 0.5	345
P84/Fe@C_60_(0.5%)	2.39 ± 0.13	113 ± 7	115 ± 4	8.9 ± 0.6	346
P84 P84/Fe@C_60_(1%)	2.39 ± 0.11	112 ± 4	97 ± 2	10 ± 1	346

**Table 2:** Thermal stability indices of membranes.

Membrane	*τ*_1_, °C	*τ*_5_, °C	*τ*_10_, °C
P84	400	466	507
P84/Fe@C_60_(0.1%)	406	472	508
P84/Fe@C_60_(0.5%)	410	476	509
P84/Fe@C_60_(1%)	400	463	503

**Table 3:** Physical properties of liquids under the study [43].

Liquid	MW	Density, g/cm^3^	Molar Volume, m^3^/mol	Boiling Point, °C	Viscosity, mPa∙s	Solubility Parameter, (J/cm^3^)^1/2^
Methanol	32.0	0.792	40.4	64.7	0.54	29.7
Methyl acetate	74.1	0.933	79.4	57.1	0.36	19.6

**Table 4:** Sorption degree of membranes.

Membrane	Sorption Degree, %	
	Methanol	Methyl Acetate
P84	7.8	17.0
P84/Fe@C_60_(0.1%)	11.6	16.8
P84/Fe@C_60_(0.5%)	11.8	16.7
P84/Fe@C_60_(1%)	12.8	16.0

**Table 5:** Comparison of transport properties of the present P84/Fe@C_60_(0.5%) membrane with literature data on the pervaporation of methanol—methyl acetate mixtures.

Membrane	Methanol in Feed, wt %	*T*, °C	Flux, kg/(m^2^ h)	Methanol in Permeate, wt %	Separation Factor	Reference
Cuprophane	19.9	45	0.453	66.3	7.9	[48]
Cuprophane	20.8	30	0.222	68.0	8.1	[48]
Pervap 2255_40	21	45	4.1	45.5	3.1	[29]
Pervap 2255_50	16	45	1.1	55.0	6.4	[29]
Pervap 2255_30	20	40	2.44	54.4	4.8	[46]
PA/ND(3%)	18	25	0.22	74.0	13.0	[47]
P84/Fe@C_60_(0.5%)	18	25	0.072	64.3	8.2	
